# An evaluation of a personalised text message reminder compared to a standard text message on postal questionnaire response rates: an embedded randomised controlled trial

**DOI:** 10.12688/f1000research.22361.1

**Published:** 2020-02-26

**Authors:** Ann Cochrane, Charlie Welch, Caroline Fairhurst, Sarah Cockayne, David J. Torgerson, OTIS Study Group

**Affiliations:** 1 York Trials Unit, University of York, York, UK

**Keywords:** SWAT, Randomised Controlled Trial, personalised, SMS text, postal questionnaire, reminder

## Abstract

**Background**: Research outcome data is commonly collected using postal questionnaires; however, poor response can introduce bias and reduce statistical power. Text messaging is simple, cost-effective, and can be customised to the individual. Personalised, reminder text messages may improve response rates.

**Methods**: A two-arm, parallel group ‘Study within a Trial’ (SWAT) was embedded within the Occupational Therapist Intervention Study (OTIS), a randomised controlled trial of a home assessment for falls prevention in older people.  OTIS participants who provided a mobile phone number were randomly allocated (1:1) to receive either a personalised text message (Title, Surname, plus York Trials Unit (YTU) text) or the standard YTU text alone, prior to receiving their four-month post-randomisation follow-up postal questionnaire. The primary outcome measure was the proportion of participants who returned the questionnaire. Secondary outcomes were: time to response, completeness of response, requirement of a reminder letter, and cost-effectiveness. Binary data were compared using logistic regression and time to response by Cox proportional hazards regression.

**Results**: A total of 403 participants were randomised: 201 to the personalised text and 202 to the standard text.  Of the 283 participants included in the final analysis, 278 (98.2%) returned their questionnaire; 136 (97.8%) for the personalised text versus 142 (98.6%) for the standard text (adjusted odds ratio 0.64, 95% CI 0.10 to 3.88, p=0.63).  The median time to response was nine days in both groups.  In total, 271 (97.5%) participants returned a complete questionnaire; 133 (97.8%) in the personalised text versus 138 (97.2%) for the standard text.  In total, 21 reminder letters were sent. The additional cost of personalised text messages was £0.04 per participant retained.

**Conclusions**: Personalised texts were not superior to standard texts in any outcome assessed in our study. Further SWATs are needed to perform a meta-analysis and obtain more evidence.

**Registration**: ISRCTN22202133; SWAT 35.

## Introduction

Evaluating strategies to improve the efficiency of conducting trials is a priority. Achieving high response rates for postal follow-up questionnaires is challenging; non-response threatens study validity through bias and reduced effective sample size ^1^. Rigorous evaluation can be achieved by undertaking a Study within a Trial (SWAT) ^2– 4^. A SWAT is a self-contained study embedded within a host trial, which aims to evaluate an intervention ^5^.

There are many strategies towards improving response to postal questionnaires including short messaging service (SMS) text prompts; however, uncertainty remains ^6, 7^ as to their effectiveness ^8– 13^. Furthermore, some evidence exists ^14^ to suggest that personalised texts, in which recipients were addressed by name, increased average payment of delinquent fines compared to non-personalised texts.

Here, we report the results of a SWAT evaluating a personalised text compared to a standard (non-personalised) text on postal questionnaire response rates in an elderly population.

## Methods

### Design

This two-arm, parallel-group, individually randomised controlled trial (RCT) was embedded within OTIS, a UK-based modified cohort RCT of occupational therapist-led home environmental assessment for the prevention of falls in older people ^15^. This SWAT was registered as part of the host trial (OTIS) registration ( ISRCTN22202133; date registered: 20.06.2016) and with the Northern Ireland Hub for Trials Methodology Research SWAT Repository ( SWAT 35; date registered: 20.02.2016).

### Participants

Eligible OTIS participants who agreed to receive text communication during participation, provided a mobile number, and were due to receive their four-month post-randomisation postal questionnaire, were randomised into this SWAT.

### Intervention

Participants received a single text four days after their four-month questionnaire was posted ( Table 1).

**Table 1.** Text message content by allocation.

Embedded trial allocation	Text message sent to participants
Personalised text	“OTIS trial: [Title, Surname of participant] you should have received a questionnaire in the post by now. Your answers are important; so please help by returning it as soon as you can. Thanks.”
Standard text	“OTIS trial: You should have received a questionnaire in the post by now. Your answers are important; so please help by returning it as soon as you can. Thanks.”

### Outcomes

The primary outcome was the proportion of participants who returned their four-month postal questionnaire. Secondary outcomes were: time to response, completeness of response, use of a reminder letter, and cost-effectiveness ( Table 2).

**Table 2.** SWAT primary and secondary outcomes.

Outcome	Definition	Type
Proportion of questionnaires returned	Proportion of questionnaires returned to York Trials Unit at four months post-randomisation.	Binary (returned/not returned)
Time to questionnaire return	Number of days elapsed between the date the questionnaire was sent to participants and the date the questionnaire was recorded as being returned to York Trials Unit. Truncated at 120 days.	Time to event (0 – 120 days)
Completeness of response	Proportion of participants returning a sufficiently complete questionnaire. A returned four month questionnaire was defined as sufficiently complete if the participant provided responses to; 1) whether they had fallen in the previous four months; 2) the extent to which they had been worried about falling; 3) all five dimensions of the EQ-5D-5L.	Binary (complete/incomplete)
Reminder letters sent	Proportion of participants sent a reminder letter (and additional blank copy of the questionnaire) due to not having returned the questionnaire within 21 days.	Binary (sent/not sent)
Cost of retaining participants at four months	Total cost per participant of texts and additional contacts.	Continuous

### Sample size

As is usual for embedded trials, no formal power calculation was undertaken ^3– 5^ as the sample size was constrained by the number of participants available in the host trial.

### Randomisation

Eligible participants (n=403) were randomised (1:1) using randomly varying blocks of four and six, stratified by OTIS trial group allocation. Allocations were generated by the OTIS trial statistician using Stata version 13.0, before being shared with the YTU data management staff responsible for the setup of the text messaging system. Eligible participants were then matched against the generated sequence in the order that they were randomised to the main trial.

### Blinding

Participants were not aware of their involvement within this SWAT; only to the OTIS trial group allocation. Study team members performing administrative, statistical or health economic roles were also not blinded, but data entry staff were.

### Ethical approval

Approvals were granted by NHS West of Scotland Research Ethics Committee 3 (ref. 16/WS/0154); the University of York, Department of Health Sciences Research Governance Committee and the Health Research Authority. Consent for the SWAT was waived by the above-named Research Ethics Committee.

### Statistical analysis

Analyses were conducted in Stata version 15.0 ^16^. Baseline characteristics are summarised descriptively ( Table 3). Binary outcomes were analysed using logistic regression, and time to questionnaire return was analysed using Cox proportional hazards regression. Time to return was truncated at 120 days allowing for the next follow-up time point (eight months post-randomisation) and illustrated using a Kaplan-Meier curve. Models were adjusted for SWAT and OTIS trial allocation. Unadjusted analyses of both binary and time to event outcomes are also presented. The costs incurred retaining participants are summarised descriptively ( Table 5).

**Table 3.** Baseline characteristics of the participants included in the analysis.

**Baseline characteristic**	Personalised texts (N = 139)	Standard texts (N = 144)	Total (N = 283)
**OTIS trial allocation, n (%)**			
Usual care	96 (69.1)	99 (68.8)	195 (68.9)
Intervention	43 (30.9)	45 (31.3)	88 (31.1)
Missing	0 (0.0)	0 (0.0)	0 (0.0)
**Age (years)**			
N	139	144	283
Mean (SD)	77.8 (6.1)	76.7 (5.7)	77.3 (5.9)
Median (1 ^st^ Q, 3 ^rd^ Q)	76.8 (72.8, 81.4)	75.5 (72.3, 80.5)	76.0 (72.7, 81.1)
**Sex, n (%)**			
Male	45 (32.4)	57 (39.6)	102 (36.0)
Female	94 (67.6)	87 (60.4)	181 (64.0)
Missing	0 (0.0)	0 (0.0)	0 (0.0)
**Taking >4 prescribed medications, n (%)**			
Yes	61 (43.9)	69 (47.9)	130 (45.9)
No	77 (55.4)	74 (51.4)	151 (53.4)
Missing	1 (0.7)	1 (0.7)	2 (0.7)
**EQ-5D-5L – Mobility, n (%)**			
No problems walking	49 (35.3)	67 (46.5)	116 (41.0)
Slight problems walking	37 (26.6)	27 (18.8)	64 (22.6)
Moderate problems walking	38 (27.3)	37 (25.7)	75 (26.5)
Severe problems walking	11 (7.9)	12 (8.3)	23 (8.1)
Unable to walk	0 (0.0)	1 (0.7)	1 (0.4)
Missing	4 (2.9)	0 (0.0)	4 (1.4)
**EQ-5D-5L – Self-care, n (%)**			
No problems washing/dressing	104 (74.8)	117 (81.3)	221 (78.1)
Slight problems washing/dressing	25 (18.0)	18 (12.5)	43 (15.2)
Moderate problems washing/dressing	8 (5.8)	7 (4.9)	15 (5.3)
Severe problems washing/dressing	1 (0.7)	1 (0.7)	2 (0.7)
Unable to wash/dress myself	0 (0.0)	0 (0.0)	0 (0.0)
Missing	1 (0.7)	1 (0.7)	2 (0.7)
**EQ-5D-5L – Usual activities, n (%)**			
No problems doing usual activities	52 (37.4)	69 (47.9)	121 (42.8)
Slight problems doing usual activities	45 (32.4)	40 (27.8)	85 (30.0)
Moderate problems doing usual activities	25 (18.0)	29 (20.1)	54 (19.1)
Severe problems doing usual activities	15 (10.8)	4 (2.8)	19 (6.7)
Unable to do usual activities	1 (0.7)	2 (1.4)	3 (1.1)
Missing	1 (0.7)	0 (0.0)	1 (0.4)
**EQ-5D-5L – Pain/discomfort, n (%)**			
No pain or discomfort	24 (17.3)	28 (19.4)	52 (18.4)
Slight pain or discomfort	55 (39.6)	60 (41.7)	115 (40.6)
Moderate pain or discomfort	43 (30.9)	44 (30.6)	87 (30.7)
Severe pain or discomfort	14 (10.1)	11 (7.6)	25 (8.8)
Extreme pain or discomfort	0 (0.0)	1 (0.7)	1 (0.4)
Missing	3 (2.2)	0 (0.0)	3 (1.1)
**EQ-5D-5L – Anxiety/depression, n (%)**			
Not anxious or depressed	78 (56.1)	91 (63.2)	169 (59.7)
Slightly anxious or depressed	37 (26.6)	39 (27.1)	76 (26.9)
Moderately anxious or depressed	15 (10.8)	8 (5.6)	23 (8.1)
Severely anxious or depressed	1 (0.7)	0 (0.0)	1 (0.4)
Extremely anxious or depressed	1 (0.7)	0 (0.0)	1 (0.4)
Missing	7 (5.0)	6 (4.2)	13 (4.6)
EQ-5D-5L – General health (0 – 100) *			
N	139	143	282
Mean (SD)	74.6 (15.6)	75.2 (17.0)	74.9 (16.3)
Median (1 ^st^ Q, 3 ^rd^ Q)	80.0 (65.0, 85.0)	80.0 (66.0, 90.0)	80.0 (66.0, 88.0)

*0-worst health you can imagine, 100-best health you can imagine

## Results

**Figure 1.**
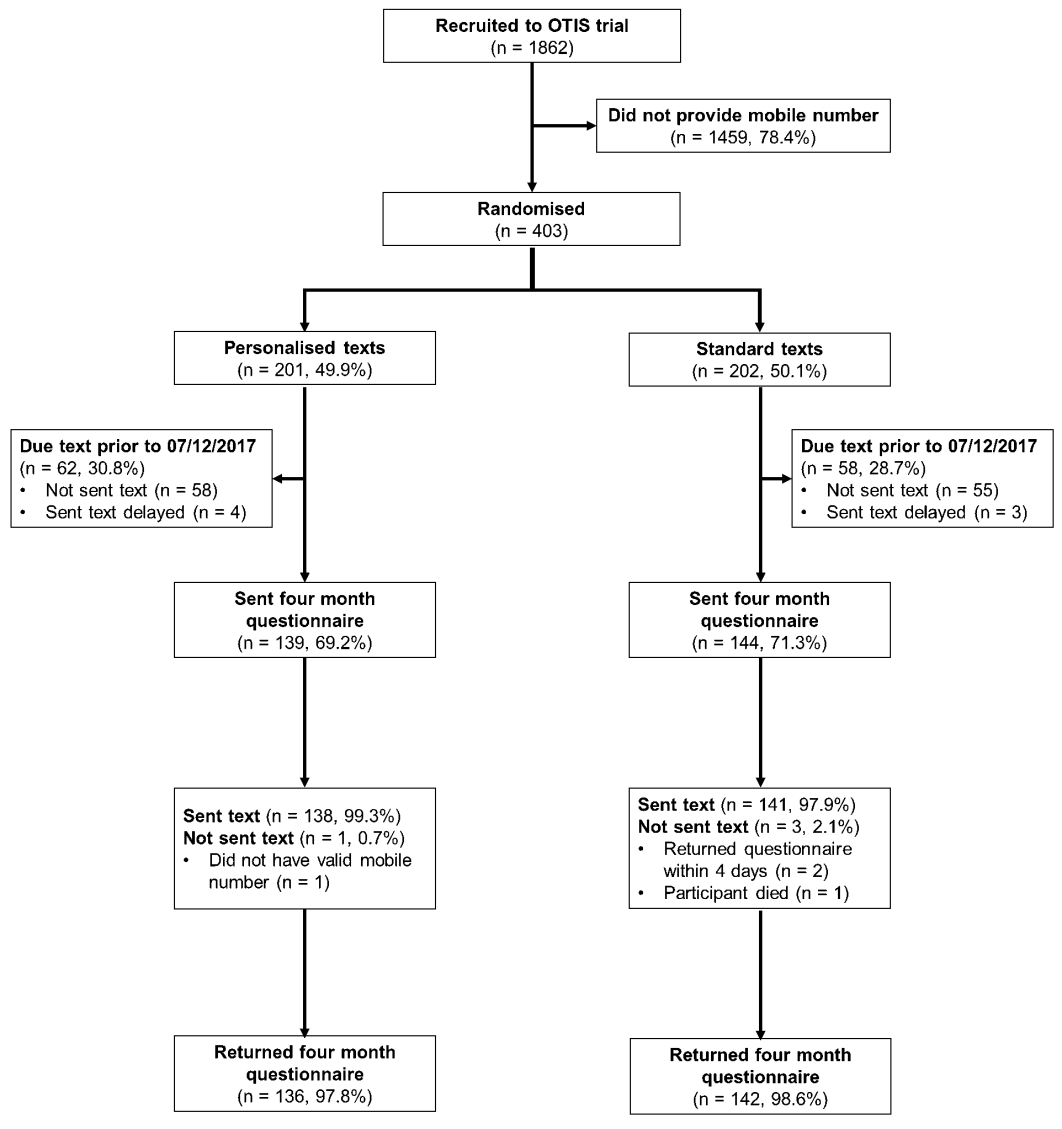
The flow of participants through the embedded trial.

Delays setting-up the text messaging system meant no texts were sent prior to 7 ^th^ December 2017. In total 120 (29.8%) randomised participants were due texts before this date. These participants are therefore excluded from the analysis. Participants (n=283) due texts on or after this date were analysed as randomised ( Figure 1).

Results are presented in Table 4. A total of 136 (97.8%) participants in the personalised text group returned their four-month questionnaire, compared with 142 (98.6%) in the standardised text group (adjusted odds ratio (OR) 0.64, 95% CI 0.10 to 3.88, p=0.63). In total, 10 personalised text participants were sent a reminder letter and 11 in the standard text arm. Of 278 returned questionnaires, 271 (97.5%) were completed: 97.8% in the personalised arm and 97.2% in the standard text arm (adjusted OR 1.29, 95% CI 0.28 to 5.89, p=0.75).

**Table 4.** Analysis of binary outcomes.

Outcome	Personalised	Standard	Analysis	OR (95% CI) (personalised/standard)	p-value
**Questionnaire returns**	136/139 (97.8%)	142/144 (98.6%)	Unadjusted	0.64 (0.11 to 3.88)	0.63
			Adjusted *	0.64 (0.10 to 3.88)	0.63
**Reminder letters sent**	10/139 (7.2%)	11/144 (7.6%)	Unadjusted	0.94 (0.38 to 2.28)	0.89
			Adjusted	0.94 (0.38 to 2.28)	0.89
**Complete questionnaires** **(returned only)**	133/136 (97.8%)	138/142 (97.2%)	Unadjusted	1.29 (0.28 to 5.85)	0.75
			Adjusted	1.29 (0.28 to 5.89)	0.75
**Complete questionnaires** **(all)**	133/139 (95.7%)	138/144 (95.8%)	Unadjusted	0.96 (0.30 to 3.06)	0.95
			Adjusted	0.96 (0.30 to 3.07)	0.95

* Primary

**Table 5.** Costs per participant of retention at four months, by allocation and overall.

Cost	Personalised texts (N = 139)	Standard texts (N = 144)	Total (N = 283)
**Cost of texts (pence)**			
Mean (SD)	9.5 (0.8)	4.7 (0.7)	7.1 (2.5)
Median (1 ^st^ Q, 3 ^rd^ Q)	9.6 (9.6, 9.6)	4.8 (4.8, 4.8)	4.8 (4.8, 9.6)
Min, Max	0.0, 9.6	0.0, 4.8	0.0, 9.6
**Cost of reminder letters (pence)**			
Mean (SD)	16.9 (60.9)	18.0 (62.6)	17.4 (61.7)
Median (1 ^st^ Q, 3 ^rd^ Q)	0.0 (0.0, 0.0)	0.0 (0.0, 0.0)	0.0 (0.0, 0.0)
Min, Max	0.0, 235.0	0.0, 235.0	0.0, 235.0
**Total costs (pence)**			
Mean (SD)	26.4 (61.0)	22.7 (62.7)	24.5 (61.8)
Median (1 ^st^ Q, 3 ^rd^ Q)	9.6 (9.6, 9.6)	4.8 (4.8, 4.8)	9.6 (4.8, 9.6)
Min, Max	0.0, 244.6	0.0, 239.8	0.0, 244.6

**Figure 2.**
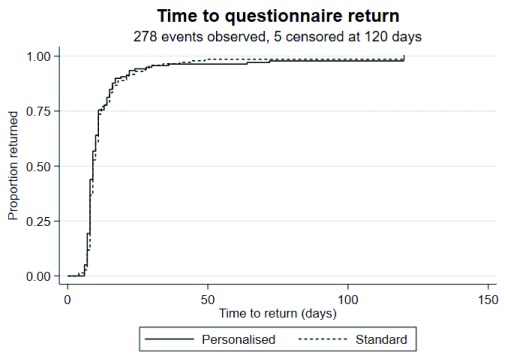
Kaplan-Meier curve for time to questionnaire return.

The median time to return was nine days in both groups. A log-rank test gave a p-value of 0.57; hence, the data provide little evidence to reject the hypothesis that the two groups have the same survival function. The Cox proportional hazards model corroborated this (hazard ratio 1.06, 95% CI 0.84 to 1.35, p=0.60) ( Figure 2). Examination of the log-log plots of the estimated survival functions, and a global test of the Schoenfeld residuals suggested the proportional hazards assumption was reasonable (p=0.52).

### Cost-effectiveness

Standard texts were 159 characters (costing £0.048), whereas personalised texts ranged from 166 to 178 characters (costing £0.096). Other costs included reminder letters and additional questionnaires posted to non-responders (£2.35 each) ( Table 5).

## Discussion

These results provide little support to the hypothesis that personalisation of texts improves postal questionnaire return rate compared to standard texts, in this population. There was also little evidence to suggest that personalisation led to quicker returns of questionnaires, improved questionnaire completion, or reduced the requirement for a reminder letter to elicit a response. The additional cost of personalised texts was £0.04 per participant retained.

### Limitations

Eligible participants who provided a mobile phone number at enrolment to the host trial (78.4%) was lower than antipated. Nearly 30% of SWAT participants had to be excluded from analysis due to problems with text automation. Furthermore, the high proportion of returned postal questionnaires in the standard text group meant only very small improvements could ever be observed or that a ceiling effect may have been reached. Thus, a large sample size would be required in order to provide strong evidence against the null hypothesis in favour of personalisation. Together, the small sample size and high baseline event rate mean this SWAT provides limited evidence for (or against) the personalisation of texts as a means to improving retention of participants.

## Conclusions

Given the uncertainty regarding the effectiveness of personalising text messages, we feel that further investigation via RCTs is warranted. Meta-analysis could be used to obtain a more precise estimate for the effectiveness of personalising texts and explore variation across different participant characteristics.

## Data availability

### Underlying data

Open Science Framework: OTIS Trial Text SWAT. https://doi.org/10.17605/OSF.IO/KH75X ^17^.

This project contains the following underlying data:

- OTIS_textswat_data (CSV). Underlying data associated with this study.
- OTIS_textswat_data (DTA). Underlying data associated with this study.
- OTIS_textswat_data_key (CSV). Key to abbreviaitons used in dataset.

### Reporting guidelines

Open Science Framework: CONSORT checklist for ‘An evaluation of a personalised text message reminder compared to a standard text message on postal questionnaire response rates: an embedded randomised controlled trial’. https://doi.org/10.17605/OSF.IO/KH75X ^17^.

Data are available under the terms of the Creative Commons Zero "No rights reserved" data waiver (CC0 1.0 Public domain dedication).
