# Supplementary material for: An evaluation of a personalised text message reminder compared to a standard text message on postal questionnaire response rates: an embedded randomised controlled trial
Source: F1000Res. 2020 Feb 26;9:154. [Version 1] doi: 10.12688/f1000research.22361.1 (PMC7194505; doi:10.12688/f1000research.22361.1)
Supplement: Peer review history

# Peer review: Reviewer response for version 1

**DOI:** 10.5256/f1000research.24670.r61887

**Published:** 2020-04-21

**Authors:** Phil Edwards

**Affiliations:** 1 Department of Population Health, London School of Hygiene and Tropical Medicine, London, UK

It has been shown previously that postal questionnaire response can be increased by using an SMS reminder (Edwards *et al.*, 2009 ^1^) and that questionnaire response can be increased by personally addressing participants by name on cover letters (Scott and Edwards, 2006 ^2^). There have, however, been few randomised trials of a personalised reminder SMS. This study is therefore a welcome addition to the literature.

 This is a report of the results of a randomised controlled trial nested within an RCT of occupational therapist-led home environmental assessment for the prevention of falls in older people. The nested trial sought to evaluate the effect on questionnaire response of a personalised text message compared to a standard (non-personalised) text in this elderly population. 403 participants who had agreed to receive text communication during participation, had provided a mobile number, and were due to receive their four-month post-randomisation follow-up postal questionnaire, were randomised into this nested trial. The proportion that responded in the personalised text message group was 97.8%, lower than the proportion that responded in the non-personalised text message group (98.6%); there was thus no evidence that the intervention had any effect on questionnaire response (p=0.63). The report is a clear and concise account of the study, citing the current literature. The authors may consider it helpful to include Pippa Scott’s study in their introduction or discussion. The study design seems appropriate and the work appears to be technically sound. The conclusions are adequately supported by the results, and the study makes a useful contribution to the data collection literature.

Is the work clearly and accurately presented and does it cite the current literature?

Partly

If applicable, is the statistical analysis and its interpretation appropriate?

Yes

Are all the source data underlying the results available to ensure full reproducibility?

Yes

Is the study design appropriate and is the work technically sound?

Yes

Are the conclusions drawn adequately supported by the results?

Yes

Are sufficient details of methods and analysis provided to allow replication by others?

Yes

Reviewer Expertise:

Evidence-based Data Collection

I confirm that I have read this submission and believe that I have an appropriate level of expertise to confirm that it is of an acceptable scientific standard.

## References

[1] : Methods to increase response to postal and electronic questionnaires. *Cochrane Database Syst Rev*.2009; 10.1002/14651858.MR000008.pub4 MR000008 10.1002/14651858.MR000008.pub4 19588449PMC8941848

[2] : Personally addressed hand-signed letters increase questionnaire response: a meta-analysis of randomised controlled trials. *BMC Health Serv Res*.2006;6: 10.1186/1472-6963-6-111 111 10.1186/1472-6963-6-111 16953871PMC1574304

# Peer review: Reviewer response for version 1

**DOI:** 10.5256/f1000research.24670.r60604

**Published:** 2020-03-11

**Authors:** Frances Shiely

**Affiliations:** 1 School of Public Health, University College Cork, Cork, Ireland

**Summary:**

 This was a SWAT within the OTIS trial, an occupational therapist-led home environmental assessment for the prevention of falls in older people in the UK. The SWAT was a two-arm, parallel group study. The primary outcome was the proportion of participants who returned the four-month follow-up postal questionnaire. Secondary outcomes were: time to response, completeness of response, requirement of a reminder letter, cost effectiveness. The conclusion was that personalised texts were not superior to standard texts in any of the outcomes assessed. This is a very well conducted study and the clarity of presentation is to be commended. 

**Answers to main headings for the review:**

1. The work is clearly and accurately presented for the most part, and it does cite the relevant literature as well as the current literature. I have added some comments/corrections below to address.
2. The study design is appropriate and the work is technically sound. Though I understand the purpose of the study was to evaluate personalised text messages versus non-personalised messages, I would have liked to see a comparison with those that received reminder letters only. Would this be possible with the current data set?
3. Sufficient details of the methods and analysis are provided for the most part but I’ve asked for a few minor issues to be addressed in the comments below.
4. The statistical analysis and its interpretation are appropriate.
5. Yes, the authors have added information on data availability.
6. The conclusions drawn are supported by the results but they are different in the abstract to the discussion section of the paper. The authors are definitive in the abstract but are less definitive in the discussion section saying the results provide “little” support. Can we really say little support? I think this should be stronger. There is no evidence to support personalised text messages in this study. I understand there were limitations but these are the findings from this study. I’d ask the authors to address the use of the term “little” in the discussion section.

 

**Further Comments/Corrections:**

- The reminder letter appears for the first time in the results section and in table 4. This needs to be detailed in the methods section. What justified a person being sent a reminder letter/why were only 21 reminder letters sent? Also, you should add the reminder letter to Figure 1. I expect that their inclusion didn’t affect the results given the small numbers and equal proportions in both groups, however, a re-run of the analysis excluding them, and a sentence to say that it didn’t affect the results.
- Rather than just present the actual costs of each type of text message in the results section under “cost-effectiveness”, can you present the findings of the analysis from both? I appreciate it’s presented in Table 5 but comment on it. 
- The outcome definition in Table 2 “proportion of questionnaires returned” should be adjusted as its current meaning does not match your intention. It is not the proportion of questionnaires returned to YTU at four months post-randomisation, it is the proportion of questionnaires returned or the proportion of questionnaires distributed at the four-month randomisation period that were returned or as you have in your main text, the proportion of participants who returned their four-month postal questionnaire. It would be helpful if you specified here how long you gave them to return it. I would add…"within x days".
- Paragraph two of the results section, "…compared with 142 in the standardised text group….” - replace standardised with standard.
- In Table 5, the heading cost of texts is misleading as it implies that more than one text was sent to each person. Change to “cost of text”.

Reviewer Expertise:

Epidemiology and Clinical Trial Methodology

I confirm that I have read this submission and believe that I have an appropriate level of expertise to confirm that it is of an acceptable scientific standard.
